# Exploring the epigenetic landscape: The role of 5-hydroxymethylcytosine in neurodevelopmental disorders

**DOI:** 10.1017/pcm.2024.2

**Published:** 2024-04-01

**Authors:** Mohamed Adil Shah Khoodoruth, Widaad Nuzhah Chut-kai Khoodoruth, Rafaa Al Alwani

**Affiliations:** 1 Department of Child and Adolescent Psychiatry, Hamad Medical Corporation, Doha, Qatar; 2 Division of Genomics and Precision Medicine, College of Health and Life Sciences, Hamad Bin Khalifa University, Doha, Qatar; 3 Communicable Disease Center, Hamad Medical Corporation, Doha, Qatar; 4 College of Science and Engineering, Hamad Bin Khalifa University, Doha, Qatar

**Keywords:** 5-hydroxymethylcytosine, autism, DNA methylation, epigenetics, neurodevelopment, precision psychiatry

## Abstract

Recent advances in genetic and epigenetic research have underscored the significance of 5-hydroxymethylcytosine (5hmC) in neurodevelopmental disorders (NDDs), such as autism spectrum disorder (ASD) and intellectual disability (ID), revealing its potential as both a biomarker for early detection and a target for novel therapeutic strategies. This review article provides a comprehensive analysis of the role of 5hmC in NDDs by examining both animal models and human studies. By examining mouse models, studies have demonstrated that prenatal environmental challenges, such as maternal infection and food allergies, lead to significant epigenetic alterations in 5hmC levels, which were associated with NDDs in offspring, impacting social behavior, cognitive abilities and increasing ASD-like symptoms. In human studies, researchers have linked alterations in 5hmC levels NDDs through studies in individuals with ASD, fragile X syndrome, TET3 deficiency and ID, specifically identifying significant epigenetic modifications in genes such as *GAD1*, *RELN*, *FMR1* and *EN-2*, suggesting that dysregulation of 5hmC played a critical role in the pathogenesis of these disorders and highlighted the potential for targeted therapeutic interventions. Moreover, we explore the implications of these findings for the development of epigenetic therapies aimed at modulating 5hmC levels. The review concludes with a discussion on future directions for research in this field, such as machine learning, emphasizing the need for further studies to elucidate the complex mechanisms underlying NDDs and to translate these findings into clinical practice. This paper not only advances our understanding of the epigenetic landscape of NDDs but also opens up new avenues for diagnosis and treatment, offering hope for individuals affected by these conditions.

## Graphical abstract

**Figure:**
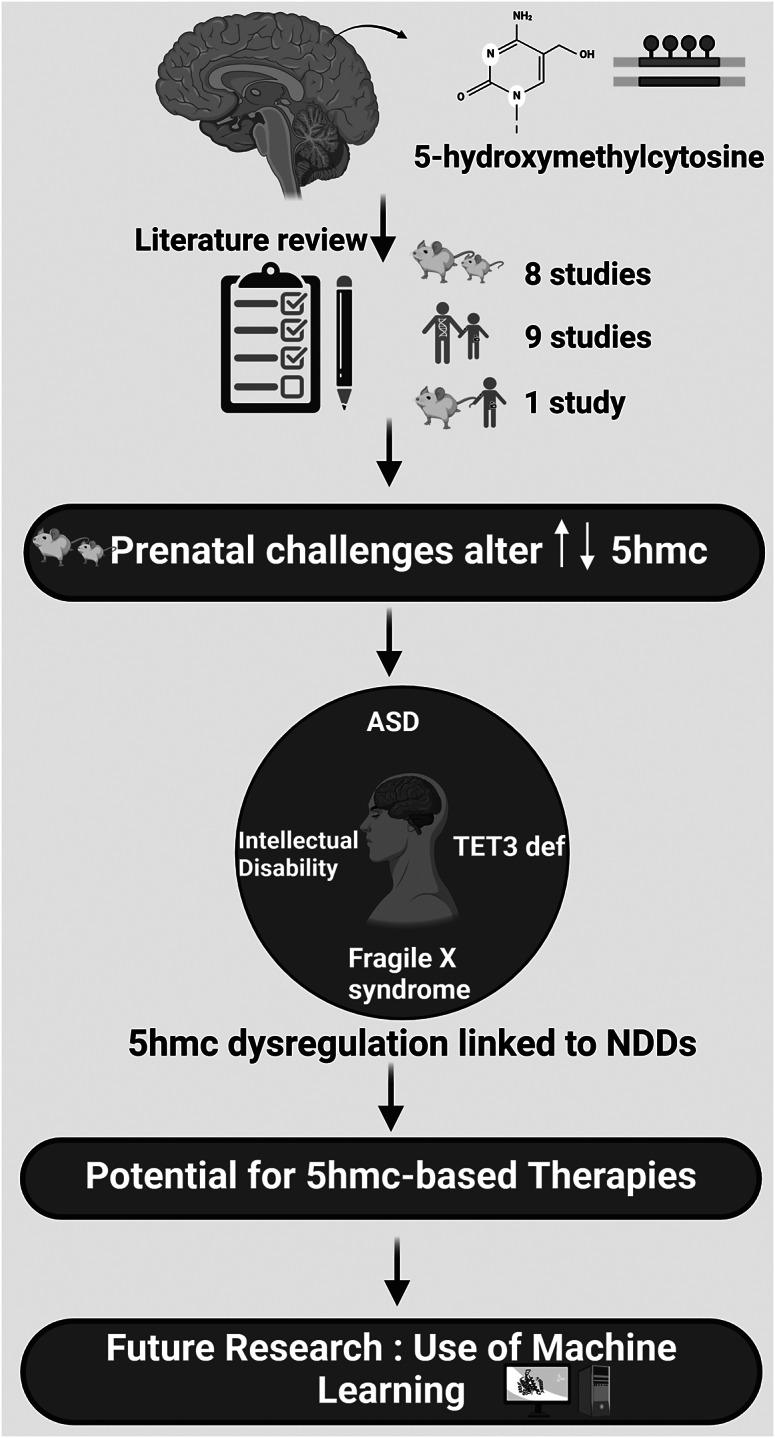

## Impact statement

This review article sheds light on the crucial role of 5-hydroxymethylcytosine (5hmC) in the understanding and future treatment of neurodevelopmental disorders (NDDs), such as autism spectrum disorder (ASD) and intellectual disability (ID). By focusing on this relatively underexplored epigenetic modification, the review highlights how environmental factors and genetic predispositions interact to influence brain development and function. By analyzing both animal and human studies, this review explains how alterations in 5hmC levels and distribution can profoundly influence neurodevelopmental outcomes. The findings have the potential to enhance diagnostic precision, enabling earlier identification and intervention for individuals at risk of these conditions. This article encourages the development of innovative therapeutic strategies aimed at modulating 5hmC dynamics, thereby offering hope for targeted effective treatments for NDDs. This paper stands to benefit a broad spectrum of stakeholders, including patients, clinicians and researchers, by offering new insights into the complex mechanisms of NDDs and fostering the development of targeted interventions. The global impact of this field is profound, offering hope for improved quality of life for individuals with NDDs and their families through earlier diagnosis, better understanding of the condition’s etiology and more effective, tailored treatments.

## Introduction

Over the past three decades, there has been an unprecedented surge in global genetic association studies aimed at identifying genetic variants implicated in the etiology of mental illnesses, including neurodevelopmental disorders (NDDs). Despite the examination of tens of thousands of cases and controls in genome-wide association studies (GWAS), none of the identified genes have demonstrated an effect size exceeding 1% (Abdolmaleky et al., 2015). Consequently, geneticists have turned their attention to the realm of epigenetics when investigating psychiatric disorders, including neurodevelopmental conditions such as autism spectrum disorder (ASD) and attention-deficit hyperactivity disorder (ADHD). Epigenetics, a term coined by Conrad H. Waddington, a prominent geneticist and developmental biologist, more than six decades ago (Waddington, 1957), refers to the factors that influence gene expression without altering the underlying DNA sequence (“on top of” genetics) (Slack, 2002). In psychiatric research, the field of epigenetics primarily explores the interplay between environmental factors and the genome (Gottesman and Shields, 1982). The epigenetic hypothesis is gaining increasing support among biological psychiatrists due to its potential to elucidate gender disparities, the clinical heterogeneity observed in presentations, and the rapid progression of psychiatric disorders (Petronis, 2001).

Epigenetic mechanisms, including DNA methylation and histone modifications, play pivotal roles in regulating gene function and have profound implications for behavioral and neuronal alterations observed in psychiatric disorders. These epigenetic modifications, akin to genetic mutations, can potentially influence gene expression and contribute to the pathogenesis of psychiatric disorders. Notably, individuals with ASD exhibit elevated expression of DNA methylation-promoting enzymes, such as DNMT1, DNMT3A and DNMT3B, in their cerebellum, indicating an overall increase in DNA methylation and hydroxymethylation (Keil and Lein, 2016).

This review aims to delve specifically into the role of 5-hydroxymethylcytosine (5hmC) and its involvement in the pathogenesis of NDDs. This review is intended to bridge a critical gap in our knowledge by focusing on the epigenetic modification 5hmC, which has been relatively understudied in the context of NDDs such as ASD and intellectual developmental disorder (IDD). The scope of this review encompasses animal and human studies, focusing on how changes in 5hmC levels and distribution correlate with neurodevelopmental conditions, thereby aiming to uncover novel insights that could pave the way for innovative therapeutic strategies and a better understanding of these complex disorders.

### The formation and maintenance of 5hmC

**Figure 1.**
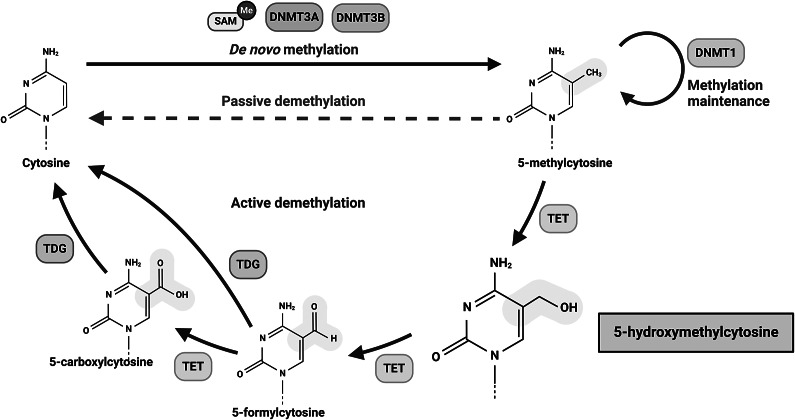
The DNA methyltransferase (DNMT) family of enzymes, DNMT1, DNMT3A and DNMT3B, catalyze the transfer of a methyl group from S-adenosyl-L-methionine (SAM) to the 5′ carbon of cytosine (C), resulting in 5-methylcytosine (5mC) (marker of gene repression). 5mC is converted back to cytosine by two possible mechanisms. One mechanism is the conversion of 5mC to 5hmC (variable marker of gene activation or repression) by the ten-eleven translocation methylcytosine dioxygenases (TET). 5hmC may then be converted to cytosine *via* subsequent enzymatic steps. A second possible mechanism involves a series of DNA replications or passive demethylation that, likely with decreased DNMT1 levels, fail to reproduce the methylation state of cytosine. Adapted from Khoodoruth and Khoodoruth (2024).

The human genome exhibits nucleotide modifications primarily through cytosine methylation and hydroxymethylation, with the latter being first observed in human Purkinje neurons in 2009 (Kriaucionis and Heintz, 2009). These modifications involve the addition of a methyl group to the 5′ carbon of cytosine, forming 5-methylcytosine (5mC), followed by the addition of a hydroxy group to the methyl group to generate 5hmC. The process of these modifications is illustrated in Figure 1. While 5mC is present in all cell types, current understanding suggests that 5hmC is predominantly restricted to the brain and early stages of development (Kriaucionis and Heintz, 2009; Tahiliani et al., 2009; Wen and Tang, 2014). However, ongoing research is continuously unveiling new insights into the distribution of 5hmC throughout the human body. Figure 1 also highlights two potential biological functions of 5hmC: its involvement in demethylation pathways and its ability to influence gene regulation through binding regulatory factors (Jin et al., 2010). Both of these hypotheses represent active areas of research, with much yet to be discovered.

### Neurodevelopmental disorders

NDDs are the first chapter and represent a prominent category in the latest edition of the Diagnostic and Statistical Manual of Mental Disorders (DSM-5-TR) (American Psychiatric Association, 2022). These disorders typically emerge during the developmental period and often manifest early in life. NDDs are characterized by deficits or variations in brain processes that give rise to impairments in personal, social, academic, or occupational functioning (Khoodoruth et al., 2023b). IDD, ASD and ADHD, among others, are encompassed within the spectrum of NDDs. Notably, extensive research has focused on elucidating the regulatory factors governing genes implicated in ASD, which affects 1 in 36 children and is characterized by impairments in social interaction and communication and restricted or repetitive behavioral patterns (Centers for Disease Control and Prevention, 2021). In contrast, despite ADHD exhibiting a community prevalence of 5% and being characterized by symptoms of hyperactivity, impulsivity and inattention that are inconsistent with developmental levels, there is a scarcity of epigenetic studies exploring this specific domain (Khoodoruth et al., 2022).

### Search criteria

**Figure 2.**
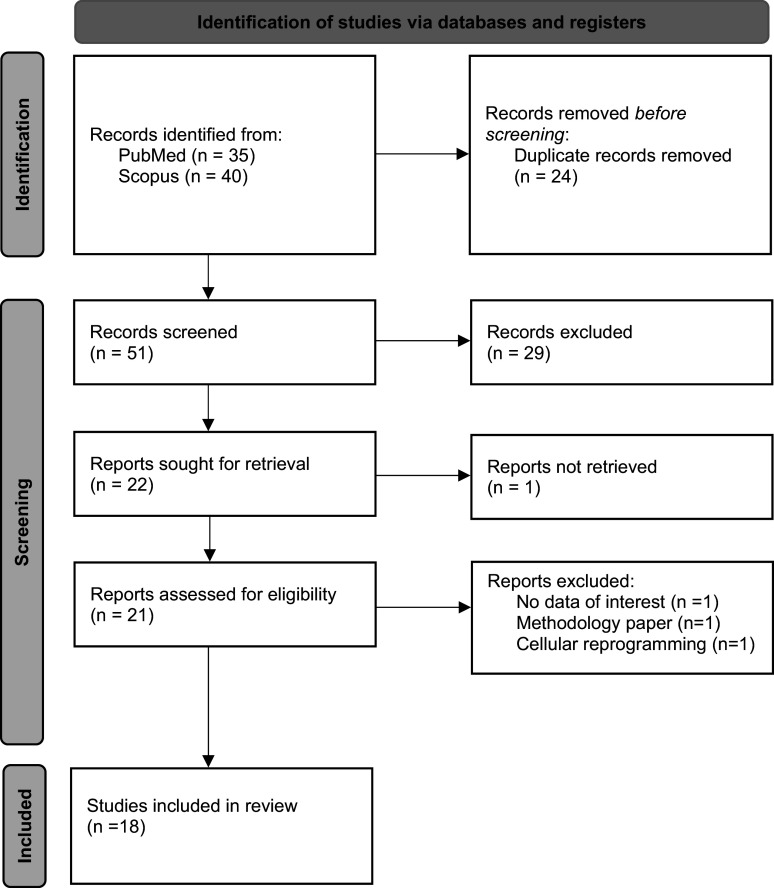
Flow Chart of Included and Excluded Studies.

Resources were retrieved from two databases, PubMed and Scopus, for this review. Initially, 75 papers were retrieved. After removing duplicates and screening titles and abstracts, 18 papers were included that were relevant to our study (Figure 2). The keywords used to search for resources in the mentioned databases were as follows: (5-hydroxymethylcytosine OR 5-hmc OR 5hmc) AND (“neurodevelopmental disorder” OR autism OR adhd OR “intellectual disability” OR “learning disability”). The included studies are summarized in Table 1.

**Table 1.** Epigenetic regulation of neurodevelopmental disorders: insights from animal and human studies on 5-hydroxymethylcytosine (5hmC)

Authors	Cases	Control	Sample	Main findings	Interpretations
*Animal studies*					
Labouesse et al. (2015)	Offspring of poly (I:C)–exposed mothers (N = 8)	Offspring of vehicle–exposed mothers (N = 8)	Medial prefrontal	Increased levels of 5hmC at two distinct regions of the *GAD1* CpG islands spanning the TSS, namely (−193 to −74 bp) and (+35 to +268 bp) 5hmC levels at the GAD2 promoter region were not affected by prenatal immune activation 5hmC enrichment at GAD1 promoter region had a negative correlation with social interaction score in both poly(I:C) model and control offspring There was no correlation between increased 5hmC levels at the *GAD2* promoter and social interaction score	Epigenetic parameters at the prefrontal GAD1 region are more involved in social interactions than those of GAD2 promoter region Maternal infection during pregnancy can cause behavioral and cognitive impairments in the long term to the offspring
Papale et al. (2015)	Animal model Autism mouse model (*Cntnap2*−/− mice)		Whole brains were extracted, striatum tissue excised	There was a genome wide disruption in 5hmC in genic region and repetitive elements at 9 weeks old *Cntnap2*−/− Annotation of DhMRs to genes showed a noticeable overlap with known ASD genes carrying the enrichment of neuronal ontological functions	The role of 5hmC in the pathogenesis of ASD will help in more understanding of the genome–wide consequence of *Cntnap2* in the future which represents an autism–like phenotype A role for 5hmC in GABAergic interneuron development
Cheng et al. (2017)	*Mecp2*–null rat Rett syndrome	WT rat cortex	Brain tissue	*MeCP2* inactivation in cultured cortical neurons led to widespread alternations of mRNA alternative splicing ChIP–seq datasets analysis showed that MeCP2–regulated exons demonstrated specific epigenetic signatures with 5hmC DNA and histone modification. H3K4me3 are enriched in down–regulated exons Compared to un–affected neurons, the H3K36me3 signature is enriched in exons up–regulated in *Mecp2*–knockdown neurons	The interaction of 5hmC with epigenetic changes in histone markers with MeCP2 regulated mRNA splicing and provided insights of the function of MeCP2–mediated mRNA splicing in the nervous system
Germundson et al. (2018)	5–8 C57BL/6 strain mice (whey protein sensitized)	5–8 C57BL/6 strain mice	Brain tissue	The immunoactivity presence of 5hmC was observed in both age groups of WP sensitized mice of both age groups in the amygdala. This indicates an epigenetic regulation	Mast cells and autism behaviors–like and its link with 5hmC
Zhang et al. (2019)	Pregnant Sprague–Dawley rats Rat offspring were subjected to hypoxic–ischemic (HI) brain damage (n = 3)	Sham procedures (n = 3)	Left cerebral temporal lobe cortex	HI injury in rats led to a significant decrease in the levels of 5hmC and the expression of Tet1 and Tet2 The study identified regions with altered DhMRs between HI and control groups, showing both increased and decreased 5hmC modifications. These DhMRs are associated with genes involved in neuronal functions and brain development, including some known CP–related genes (*Notch1*, *Slc16a2*, *Dmd*, *Grin2b*)	A disruption in epigenetic regulation following HI injury could contribute to CP pathogenesis
Papale et al. (2022)	Animal model – mice adult *Cntnap2* heterozygous mice (*Cntnap2*+/−; lacking behavioral or neuropathological abnormalities) subjected to a prenatal stress		Striatum and hippocampus	Prenatally stressed *Cntnap2*+/− female mice showed autism–like repetitive behaviors and were socially less interactive Genomic profiling revealed disruptions in 5hmC levels in hippocampal and striatal which are correlated to altered transcript levels of genes linked to phenotypes such as *Reln*, *Dst*, *Trio* and *Epha5* A mechanistic role for 5hmC in gene regulation	Their data support gene–by–environment hypotheses for the origins of mental illness It shows that 5hmC has a functional role in a gene interaction model that results the alteration of neurodevelopmental behaviors that are sex–specific
Cao et al. (2022)	7 male mice model		4 brain areas prefrontal cortex (PFC), amygdala, hippocampus and hypothalamic	CMA mice showed abnormal behaviors that were autism–like, such as less social interaction and self–grooming repetitive behavior The results provide evidence that 5–hmC may have a role in allergy–induced autism–like behavior	mTOR signaling pathway and *Lactobacillus* might be involved in regulating gut–immune–brain axis in the intestinal, immunological and autism–like symptoms
Bhatia et al. (2022)	Young (2–3 months) and adult (8–12 months) *OGG1* knockout mice	3 genotypes (+/+, +/−, −/−)	Whole brain: frontal cortex, striatum, cerebellum, hippocampus	In young male *Ogg1* −/− mice, 5mC levels significantly decreased by approximately 60% compared to *Ogg1* +/+ mice, while no significant effect was observed in adult males or females. Adult male brains exhibited lower 5mC levels than young males, with a similar but smaller reduction in adult females A nonsignificant trend of decreased 5hmC levels was observed in young *Ogg1* −/− brains compared to *Ogg1* +/+ brains, affecting both males and females. In adult +/+ brains, 5hmC levels were nonsignificantly lower than in their young counterparts for both sexes, with no specific trends in *OGG1* −/− brains Significantly lower 5mC levels in the cerebellum of female *Ogg1* −/− compared to *Ogg1* +/+ brains. Female *Ogg1* +/+ mice had nearly twice the 5–mC levels of male +/+ mice, while *Ogg1* −/− females showed similar levels to male mice. Similar trends, although not significant, were observed for 5hmC levels. In the hippocampus, 5mC and 5hmC levels were lower in male *Ogg1* −/− compared to +/+ brains, but no significant differences were observed in females. Male *Ogg1* +/+ mice had significantly higher 5mC and 5hmC levels compared to female +/+ mice	These findings highlight the complex relationship between *OGG1* genotypes and the levels of 5mC and 5hmC in the brain, which vary depending on age, sex, and the specific epigenetic mark being examined
*Human studies*					
Wang et al. (2012)	One human fetal sample	One female and one male adult	Cerebellar DNA samples	Increase in total 5hmC levels in the adult human cerebellum compared to the fetal stage, suggesting an important role of 5hmC in cerebellum development	The enrichment of 5hmC in *FMRP* target genes and the association of DhMRs with ASD candidate genes suggest that 5hmC dysregulation may contribute to the molecular pathogenesis of NDDs
Zhubi et al. (2014)	10 ASD 9 M, 1 F Age range: 15–56	10	Cerebellar cortex	A significant increase in *TET1* expression and an enrichment in the level of 5hmC, but not 5mC, at the promoters of *GAD1* and *RELN* in ASD when compared with controls. In addition, there was increased *TET1* binding to these promoter regions A marked and significant increase in MeCP2 binding to the promoter regions of *GAD1* and *RELN*	An increase of 5hmC (relative to 5mC) at specific gene domains boosts the binding of MeCP2 to 5hmC and decreases expression of the corresponding target genes in ASD cerebella
James et al. (2014)	13 ASD	13	Cerebellar cortex	5hmC was noticeably higher in ASD cases and accompanied by increases in *DNMT3A and DNMT3B*, *TET1 and TET3 genes*, and in 8–oxo–dG expressions Within the *EN–2* promoter, there was a significant positive correlation between 5hmC and *EN–2* gene expression A decrease in MeCP2 binding to the same 5′ promoter region that contained elevated levels of 5hmC in ASD to controls	An increase in 5hmC and decrease in both MeCP2 and histone H3K27me3 binding may modify local chromatin conformation to facilitate enhancer binding and the persistent upregulation of *EN–2* gene expression in the postnatal autism cerebellum
Brasa et al. (2016)	Eight FXS (>200 CGG repeats) male patients, 12–45 years old	Seven controls	PBMCs	DNA methylation levels of *FMR1* positively correlated with FXS disease severity, while an inverse correlation was found between 5hmC levels and disease severity	The potential of combining 5mC and 5hmC measurements at the *FMR1* locus for improved clinical diagnostics and patient stratification
Esanov et al. (2016)	12 FXS and FXTAS (55–200 CGG) patients	Six healthy controls	PM brain tissues	In brains of FXS patients with full mutations, there is a significant increase in 5hmC levels at the *FMR1* promoter compared to pre–mutation carriers and unaffected controls	The importance of 5hmC in the epigenetic regulation of the FMR1 gene in FXS
Zhubi et al. (2017)	8 ASD	10 CON	Frontal cortex	This study is a continuation of Zhubi et al. (2014) previous study on postmortem CB of ASD patients Compared with an increase in 5hmC content in ASD CB, there was no change in 5hmc in ASD FC relative to controls. However, in both cases there was an increase in the 5hmC/5mC ratio. The increase in ratio in CB was due to increased levels of 5hmC. Nonetheless, the increase in ratio in FC was driven by a decrease in 5mC	Increased MECP2 binding to the RELN, GAD1 and GAD2 promoters, with reduced amounts of 5mC and unchanged amounts of 5hmC present in these regions, suggests the possibility that DNMT1 interacts with and alters MeCP2 binding properties to selected promoters
Cheng et al. (2018)	17 ASD Young group (<=18) 4 M, 1F Middle group 18–35 yo: 6 M Old group > = 35: 4 M,2F	19 Young group Middle group Old group	Genomic DNA isolated from the PM cerebellum	DhMRs that are exclusive to the young group Pathway and disease association analyses showed that the intragenic DhMRs identified in the young group were in the genes involved in cell–cell communication and neurological disorders DhMRs could potentially have *cis* functions that associate with ASD and IDs About 40% of the intergenic DhMRs overlapped with predicted enhancers	The dynamic change of 5hmC could play essential roles in early neurodevelopment and ASD pathogenesis
Corley et al. (2019)	Sub–analysis of 5 young ASD pts	5 typically developing samples	34 frozen PM brain tissue specimens	Depletion of 5mC at transcription start sites and CpG islands Compared to that of typically developing individuals, 5mC in ASD brain was hypomethylated at TSS, gene bodies and canonical exons 5hmC contrasted the distribution of 5mC and was increased at TSS, gene bodies and canonical exons 5hmC was depleted at TSS and CpG islands in the SVZ from typically developing brain	This study suggests active demethylation or unresolved poised sites in the genome associated with ASD
Beck et al. (2020)	11 patients of *TET3* deficiency in eight families	N/A	The study utilized a cell–culture system to demonstrate that most *TET3* genetic variants in affected individuals have reduced efficiency in converting 5mC to 5hmC, suggesting a hypomorphic function that could contribute to disease pathogenesis	*TET3* deficiency was identified as a Mendelian disorder associated with a range of neurodevelopmental and physical conditions, such as IDD and growth discrepancies, caused by mutations in the *TET3* gene that disrupt DNA demethylation Functional assessments revealed that the *TET3* variants from affected individuals, especially those in the catalytic domain, lead to diminished enzymatic function and decreased production of 5hmC	*TET3* deficiency, alongside other Mendelian epigenetic disorders, exhibits significant similarities in symptoms, such as IDD, emphasizing common underlying disease processes
*Mixed models*					
Shpyleva et al. (2014)	15 humans 10 mice (*BTBR T+tf/J* mice)	15 humans 10 mice	Mouse and human PM CB samples	DNA isolated from the CB of *BTBR T+tf/J* mice and from human PM CB of individuals with ASD, are both characterized by an increased levels of 8–oxodG, 5mC and 5hmC The increase in 8–oxodG and 5mC content was associated with a markedly reduced expression of the 8–oxoguanine DNA–glycosylase 1 (*Ogg1*) and increased expression of *DNMT3A* and *DNMT3B* A rise in the level of 5hmC occurred without changes in the expression of *TET1* and *TET2* genes, but significantly correlated with the presence of 8–oxodG in DNA	This study highlights the interplay among Ogg1 gene, 8–oxydG, 5mC and 5hmC

ASD, autism spectrum disorder; CB, cerebellum; CGG, cytosine–guanine–guanine; CMA, cow milk allergy; CP, cerebral palsy; CpG, 5’—C—phosphate—G—3′; DhMRs, differentially hydroxymethylated regions; DNMT3A and DNMT3B, de novo methyltransferases 3A and 3B; EN-2, engrailed-2; F, female; FC, frontal cortex; ID, intellectual disability; FGX, fragile X syndrome; FMR1, fragile X mental retardation 1 gene; FMRP, fragile X mental retardation protein; FXTAS, fragile X-associated tremor/ataxia syndrome; GAD1, glutamic acid decarboxylase 67; GAD2, glutamic acid decarboxylase 65; IDD, intellectual developmental disorder; M, male; MeCP2, methyl-CpG binding protein 2; mTOR, mammalian target of rapamycin; NDDs, neurodevelopmental disorders; OGG1, oxoguanine glycosylase 1; PBMC, peripheral mononuclear blood cells; PFC, prefrontal cortex; PM, postmortem; SVZ, subventricular zone; TET, ten-eleven translocase; RELN, reelin; WP, whey protein; 5hmC, 5-hydroxymethylcytosine; TSS, transcription start sites; 5mC, 5-methylcytosine; 8-oxo-dG, 8-oxo-deoxyguanosine.

## Results

### Animal studies

Maternal infection during pregnancy has been identified as a potential risk factor for developing NDDs in offspring (Atladóttir et al., 2010). Labouesse et al. (2015) employed a well-established mouse model of prenatal immune challenge by administering viral mimetic poly(I:C) to pregnant mice. This immune activation during pregnancy resulted in elevated levels of both 5mC and 5hmC in the prefrontal cortex, specifically in the promoter region of *GAD1.* Additionally, increased levels of 5mC were observed in the promoter region of *GAD2* following early-life challenges. These prenatal infection-induced modifications were associated with impairments in social interaction, challenges in working memory and abnormal cognitive abilities (Labouesse et al., 2015).

Papale et al. (2015) conducted a study involving three mouse models of autism (*Cntnap2*−/−) and three age-matched control littermates, sacrificing them at 9 weeks of age. A genome-wide sequencing approach was employed on the striatal tissue, revealing a widespread distribution of 5hmC in the *Cntnap2*−/− mice, particularly within genic regions and repetitive elements. Notably, a significant overlap was observed between neuronal projection morphogenesis and the known ASD genes, suggesting an enrichment of neuronal ontological functions (Papale et al., 2015). Additionally, Zhang et al. (2019) highlighted a significant reduction in 5hmC and Tet proteins, global alterations in 5hmC distribution and the identification of DhMRs associated with Cerebral Palsy (CP)-related genes, such as *Notch1*, *Slc16a2*, *Dmd* and *Grin2b*, indicating disrupted epigenetic regulation and potential impacts on gene expression critical to CP pathogenesis.

Amir et al., 1999 have demonstrated that mutations in the X-linked gene methyl CpG binding protein 2 (*MeCP2*) lead to Rett syndrome, a severe NDD. Additionally, duplications of genomic segments of the *MeCP2* gene have been associated with autistic features in humans (Mellén et al., 2012). In a study by Cheng et al. (2017), mass spectrometry analysis was employed to identify *MeCP2*-associated proteins involved in mRNA splicing, utilizing samples from *MeCP2*-null rat brains. The functional analysis of ChIP-seq datasets provided insights into the interaction between *MeCP2*, 5hmC and epigenetic changes in histone markers, highlighting the role of *MeCP2* in regulating mRNA splicing within the nervous system (Cheng et al., 2017).

To investigate the potential influence of food allergy and inflammation on behavior and mast cell accumulation in the brain, Germundson et al. (2018) conducted a study utilizing mouse models of milk allergy with bovine milk whey proteins (WP) as the allergen. Male and female mice from different age groups (4 weeks and 10 months) were sensitized to the allergen for 5 weeks, followed by oral challenges for three consecutive days before sacrifice. The researchers examined various factors, including epigenetic DNA modifications in the brain. The results revealed no apparent behavioral differences in female mice. However, WP-sensitized male mice exhibited reduced digging activity compared to sham males in both age groups. Additionally, a noticeable difference in 5hmC immunoreactivity was observed in the amygdala of both age groups of WP mice, suggesting an involvement of epigenetic regulation (Germundson et al., 2018). Similarly, Cao et al. (2022) suggested an association between food allergy (cow milk allergy) and an increase in ASD-like symptoms and levels of 5hmC. Results showed that mice with cow milk allergy showed autistic-like symptoms and behaviors. There was an increase in the 5hmC biomarker in the hypothalamus as well.

In a more recent paper by Papale et al. (2022), the brain tissue of ASD mouse models (*Cntnap2*) that were projected to prenatal stress was tested. The sample lacked behavioral or neuropathological abnormalities. Genomic profiling of DNA samples from hippocampal and striatal showed disruptions in 5hmC levels. The result suggests a role of 5hmC in gene regulation and possible associations with mental illness and behavioral changes.

### Human studies

Nine articles, which included human subjects with ASD, FXS, *TET3* deficiency and ID investigating 5hmC as an epigenetic marker in NDDs, were included in this review (Table 1). This shows the growing interest of geneticists and biological psychiatrists in exploring the role of environmental factors as epigenetic mechanisms, including DNA methylation/demethylation, in the development of NDDs.

In 2012, a study by Wang et al. (2012) unveiled significant insights into 5hmC dynamics during human cerebellum development. It demonstrated a substantial increase in 5hmC levels from fetal to adult stages, underscoring its pivotal role in brain maturation. Remarkably, 5hmC was found to be preferentially enriched in gene exons and untranslated regions (UTRs), pinpointing specific genomic areas influenced by this modification throughout development. The discovery of both fetus-specific and adult-specific DhMRs, especially in genes regulated by fragile X mental retardation protein (FMRP) and those linked with autism, suggested a widespread impact of 5hmC on neurodevelopment. These findings highlight the potential of dysregulated 5hmC in contributing to the molecular pathogenesis of NDDs. Furthermore, the observation that fetus-specific DhMRs retain epigenetic memories of embryonic stem cells implied these regions carry developmental epigenetic information crucial for cerebellum development gene expression patterns. The association of DhMRs with ASD candidate genes further supported the notion that 5-hmC dysregulation could be a contributing factor to NDDs, offering novel perspectives for understanding the epigenetic mechanisms underlying brain development and its related disorders.

Zhubi et al. investigated the function of 5hmC in the transcriptional regulation of ASD candidate genes, specifically *GAD1* and *RELN*, in the cerebellum and frontal cortex of individuals with ASD (James et al., 2014; Zhubi et al., 2014, 2017). Their findings revealed an enrichment of 5hmC at the promoters of *GAD1* and *RELN* in the cerebellar cortex of individuals with ASD. Additionally, there was a significant increase in the binding of MeCP2 and TET1 to the promoters of *GAD1* and *RELN* (Zhubi et al., 2014). In fact, Mellén et al. (2012) characterized MeCP2 as a significant binding protein for 5-5hmC in the central nervous system (CNS). MeCP2 was found to enhance gene transcription upon binding to 5hmC, and conversely, it exhibited a repressive effect when binding to DNA containing 5mC. These results suggest that elevated levels of 5hmC, relative to 5mC, at specific gene regions enhance the binding of MeCP2 to 5hmC, thereby reducing the expression of the target genes in the cerebellum of individuals with ASD. Notably, alterations in MeCP2 levels due to gene deletions or mutations are implicated in Rett Syndrome, a syndromic NDD with prominent features of autism (American Psychiatric Association, 1994; Chahrour and Zoghbi, 2007). MeCP2 plays a critical role in regulating synaptic and neuronal plasticity and developing motor skills, cognitive function and social behavior (Ebert and Greenberg, 2013). Furthermore, both *RELN* and *GAD1* have been implicated in the pathophysiology of ASD (Zhubi et al., 2017).

James et al. (2014) have also shown a significant increase in 5hmC levels in the cerebellum of individuals with autism, accompanied by elevated expression of *de novo* methyltransferases DNMT3A and DNMT3B, ten-eleven translocase genes *TET1* and *TET3*, and increased content of 8-oxo-deoxyguanosine (8-oxo-dG), an indicator of oxidative DNA damage. They further demonstrated a positive correlation between 5hmC content and the engrailed-2 (*EN-2*) gene expression within the *EN-2* promoter. The *EN-2* homeobox transcription factor plays a crucial role in Purkinje cell maturation, normal cerebellar patterning and development and has been implicated in ASD in multiple studies (Benayed et al., 2009; Cheng et al., 2010).

Cheng et al. (2018) conducted a study investigating 5hmC alterations in postmortem brains of individuals with ASD across different age groups. They identified differentially hydroxymethylated regions (DhMRs) specifically in the young group (age ≤ 18), while no significant DhMRs were observed in the groups over 18 years of age. These findings suggest that 5hmC alterations are associated with ASD, particularly during early development, and may contribute to the pathogenesis of the disorder. Pathway and disease association analyses further revealed that the intragenic DhMRs were enriched in genes involved in cell–cell communication and neurological disorders. Notably, many ASD risk genes play crucial roles in synapse development, which is essential for communication between brain cells (Ebert and Greenberg, 2013).

Corley et al. (2019) conducted a study focusing on the interindividual variability in DNA methylation levels among individuals with ASD by examining postmortem subventricular zone (SVZ) tissue specimens from ASD cases and control subjects. The SVZ, which is implicated in ASD pathology, serves as a significant neurogenic niche in the mammalian brain, housing neural stem cells that undergo proliferation, differentiation and migration to form the neocortex during prenatal human brain development. The study revealed significantly lower levels of DNA methylation in ASD cases than controls at differentially methylated loci (DML) in both young and middle-aged groups. Specifically, ASD brain samples exhibited hypomethylation at transcription start sites (TSS), gene bodies and canonical exons, indicating a global hypomethylation pattern in young individuals with ASD compared to typically developing individuals. In contrast, 5hmC levels were increased at TSS, gene bodies and canonical exons in ASD cases while being depleted in control samples. These findings suggest an active demethylation process in the genome associated with ASD and underscore the crucial role of 5hmC-mediated epigenetic modifications in the pathogenesis of this NDD.

Beck et al. (2020) identified *TET3* deficiency as a novel Mendelian disorder of DNA demethylation, affecting 11 patients across eight families, characterized by intellectual disability (ID), developmental delay and growth anomalies. Through analysis of mono-allelic and bi-allelic pathogenic variants, primarily within *TET3*’s catalytic domain, the study elucidated the enzyme’s vital role in 5hmC production and neurodevelopment, while underscoring the phenotypic commonalities with other epigenetic disorders, thereby advancing our understanding of the epigenetic regulation mechanisms involved in human development and disease.

Esanov et al. (2016) further elucidated the role of 5hmC in the epigenetic regulation of the *FMR1* gene in FXS, revealing significant increases in 5hmC levels at the *FMR1* promoter in full-mutation FXS patient brains compared to pre-mutation carriers and controls, with this enrichment being neuron-specific. Cellular models, including fibroblasts, lymphocytes and induced pluripotent stem cell (iPSC)-derived neurons, failed to recapitulate the 5hmC enrichment observed in primary neurons, suggesting they do not fully mimic the epigenetic landscape of FXS. Additionally, 5hmC and 5mC distributions were distinct between neuronal and non-neuronal DNA fractions, indicating cell-type-specific epigenetic regulation. The study further demonstrates that neither cell-cycle progression nor neuronal maturity affects 5hmC levels at the *FMR1* promoter, and TET enzyme expression levels do not differ significantly between FXS-derived neuronal models and primary neurons, suggesting that the lack of 5hmC enrichment in cellular models is not due to TET dysregulation. These findings highlight the critical role of 5hmC in the *FMR1* gene’s epigenetic regulation in FXS and point to the need for future therapeutic strategies targeting this pathway to restore FMR1 expression while also indicating that current cellular models may not fully capture the disease’s epigenetic nuances.

## Mixed models

Shpyleva et al. (2014) conducted a study examining DNA from the cerebellum of *BTBR T+tf/J* mice, a relevant mouse model of autism and postmortem cerebellum samples from individuals with ASD. Their findings revealed elevated levels of 8-oxo-deoxyguanosine (8-oxo-dG), 5mC and 5hmC in both mouse and human samples, corroborating earlier observations by James et al. (2014). The study further emphasized the interplay between reduced expression of 8-oxoguanine DNA-glycosylase 1 (*Ogg1*) and 5hmC in the pathogenesis of ASD.

*Ogg1*, highly expressed in the brain, protects neurons against oxidative DNA damage during development and various pathological conditions (Wong et al., 2008; Liu et al., 2011). The absence of *Ogg1* in the brain leads to various cellular and molecular events, including increased apoptosis and abnormal neuronal connectivity (Wong et al., 2008), key pathomorphological features of autism. In fact, Bhatia et al. (2022) explored the involvement of OGG1, using *OGG1* knockout mice, in brain development and its role in repairing DNA lesions induced by reactive oxygen species. Their findings demonstrate that young *Ogg1* knockout mice displayed sex- and gene-specific DNA damage, reduced DNA methylation marks, elevated cerebellar calbindin levels, impaired hippocampal function, altered body weight and various behavioral abnormalities, highlighting the significance of OGG1 in normal brain development through its potential functions in DNA repair and epigenetic regulation, with implications for NDDs.

## Discussion

### Ethical concern

In the papers mentioned in the human studies section, dissection of postmortem brains (cerebellum) of children under the age of 18 was required to be done to profile genome-wide distribution of 5hmC. There have been ethical concerns about brain banks, as many controversial concerns have arisen.

Everyone has the right to autonomy over their body per the Convention on Human Rights and Biomedicine (Hendriks, 1997). Therefore, pursuing informed consent for removing brain tissue postmortem requires consent for organ donation before death. In some cases, patients such as ASD patients are considered vulnerable. They often do not have autonomy over their bodies to make that decision, so such decisions are made by their caretakers, which raises questions about the adequacy of surrogate decision-making in capturing the donor’s wishes.

Additionally, there are no international laws about postmortem body organs and tissue donation to legalize and regulate it worldwide in a harmonized manner (Huitinga et al., 2019). To mitigate these ethical dilemmas and enhance the ethical integrity of research involving postmortem brain dissection, international collaboration should advocate for and participate in international efforts to develop harmonized legal and ethical standards and laws for postmortem donation and research. Moreover, more transparency and public engagement are needed in the future to educate people who are considering donating to brain banks on the importance of postmortem brain tissues in research, explain the ethical aspects and make sure consent forms reflect all those aspects to ensure the informed decision of donors and caretakers of vulnerable donors.

### Future perspectives

Studies showed that 5hmC is critical in brain development and neurological disorders. 5hmC-mediated pathways are essential for gene regulation (Cheng et al., 2015). Moreover, it is an intermediate in the DNA demethylation process. This finding is crucial as it will help researchers and professionals better understand the role of 5hmC on neurodevelopment disorders, and it could accelerate the research to find novel treatments or even come up with preventative measures to minimize the effect of neurodevelopment disorders.

Most studies included in this review, if not all, utilized ASD as the prototypical NDD. However, other NDDs, such as ADHD, IDD, communication disorders, learning disorders and tic disorders, warrant further epigenetic investigations. These disorders also have a profound negative impact on children’s social, psychological and academic functioning.

Moreover, most investigations focused on analyzing genome-wide DNA hydroxymethylation changes in brain samples from the cerebellum and frontal cortex. This choice of regions was motivated by the observed positive correlation between levels of 5hmC and cerebellum development in previous studies. It would be interesting to explore the enrichment of 5hmC in other brain regions, such as the parietal, temporal and occipital lobes.

Animal studies that model ASD-like symptoms offer crucial insights, yet it is imperative to distinguish between the simulation of specific behaviors and the broader scope of NDD research. This differentiation is essential for advancing our comprehension and treatment strategies for ASD and other NDDs within a comprehensive neurodevelopmental context. While rodents, particularly mice, are frequently utilized for ASD modeling due to their cost-effectiveness and sophisticated genetic manipulation capabilities, they lack the sulcus and gyrus structures present in the human brain, posing a significant limitation to their translational relevance (Li et al., 2021). Emerging technologies, including single-cell sequencing, multiphoton in vivo imaging and gene editing, represent promising advancements in unraveling the pathogenesis of NDDs (Li et al., 2021). These technologies hold the potential to overcome existing limitations by providing deeper insights into the cellular and molecular complexities underlying these disorders.

Increasing evidence is shedding light on the connection between inflammation and neuropsychiatric disorders, highlighting the significant impact of immunological responses on neurological health. Notably, the association of Pediatric Autoimmune Neuropsychiatric Disorders Associated with Streptococcal Infections (PANDAS) with conditions such as Tourette Syndrome and obsessive-compulsive disorder is well-known (Khoodoruth et al., 2023a). Furthermore, research has revealed the intricate interactions among the redox system, autophagy and the Nrf2 pathway in the pathogenesis and progression of neuropsychiatric disorders (Calabrese et al., 2007, 2010, 2016; Scuto et al., 2019). These findings underscore the pivotal role of oxidative stress and inflammation in NDDs and point to the promising therapeutic potential of targeting these pathways.

Given the limited options for pharmacological intervention in managing challenging behaviors in NDDs—where only risperidone and aripiprazole have been approved for autism-related irritability (Cohen et al., 2013)—there is a compelling need for innovative therapeutic approaches. In this context, epigenetic therapies, such as DNMT modifiers, emerge as promising candidates. Drawing on their successful application in oncology (Leone et al., 2003), these therapies hold significant promise for precision medicine in NDDs, offering new hope for addressing these complex conditions with more targeted and effective interventions.

Research has shown that machine learning can offer new insights into the intricate ways epigenetic changes affect our genes, as it has great potential to advance our understanding of the complex relationships between epigenetic modifications and gene expression. Some research highlights the importance of artificial intelligence in advancing our understanding of epigenetic modifications and their potential implications in disease diagnosis and therapy. Liu et al. (2020) discuss the important role of RNA 5hmC modification in various biological processes, including NDDs. However, identifying 5hmC sites in RNA is expensive and challenging. Therefore, the authors developed a computational protocol called iRNA5hmC, which uses machine learning to predict RNA 5hmC sites. The researchers found that the proposed feature representations were more accurate at distinguishing true 5hmC sites from non-5hmC sites compared to existing feature descriptors. This could potentially increase the efficiency of exploring 5hmC sites and their association with NDDs for scientists when exploring connection between 5hmC sites and NDDs such as ASD, encouraging more opportunities for research in this area.

In another study, Pavlovic et al. (2017) developed a machine learning framework called DIRECTION to predict and characterize DNA methylation and hydroxymethylation in mammalian genomes, providing a cost-effective and efficient alternative to existing methods. The paper presents a machine-learning framework that predicts and characterizes DNA methylation and hydroxymethylation in mammalian genomes. The framework was tested on human embryonic stem cells and neural progenitor cells, and it accurately predicted DNA methylation and hydroxymethylation, demonstrating the potential for large-scale reconstruction of epigenetic maps in mammalian model systems.

As we look to the future, enhancing machine learning algorithms to better predict 5hmC patterns—and possibly other epigenetic modifications—becomes crucial. Such advancements could provide deep insights into the role of epigenetics in NDDs. Importantly, machine learning models that accurately predict epigenetic changes hold the promise for clinical application, offering new avenues for diagnosis and treatment. By identifying specific epigenetic markers linked to disorders such as ASD, these models could pave the way for personalized medicine, enabling tailored treatment plans based on an individual’s epigenetic makeup for more effective outcomes.

## Conclusion

In summary, this review article has provided an overview of the current literature investigating the involvement of 5hmC in the development of NDDs, underscoring its diagnostic and therapeutic importance. This emphasis is particularly critical given the current absence of definitive clinical laboratory tests for the diagnosis and management of NDDs. The identification of 5hmC as a prominent epigenetic mark in the brain has expanded our understanding of the intricate mechanisms governing neurogenesis and the emergence of complex behavioral disorders. NDDs entail a multifaceted molecular pathogenesis encompassing a range of genomic, epigenomic, proteomic, metabolic and physiological alterations. Therefore, gaining comprehensive insights into the molecular processes of NDDs using advanced machine learning algorithms, whenever feasible, is crucial for effective clinical and personalized management and prevention strategies.

## Data Availability

The authors confirm that the data supporting the findings of this study are available within the article.
