# Supplementary material for: Exploring the epigenetic landscape: The role of 5-hydroxymethylcytosine in neurodevelopmental disorders
Source: Camb Prism Precis Med. 2024 Apr 1;2:e5. doi: 10.1017/pcm.2024.2 (PMC11062787; doi:10.1017/pcm.2024.2)
Supplement: Peer review history

# Editor report: Recommendation: Exploring the epigenetic landscape: The role of 5-hydroxymethylcytosine in neurodevelopmental disorders — R0/PR2

**DOI:** 10.1017/pcm.2024.2.pr2

**Affiliations:** Oxford University, United Kingdom of Great Britain and Northern Ireland

Following review by three independent experts in the field, I agree that this review addresses a relevant question and summarises important findings. However, there are also significant weaknesses.First, the scope needs to be more clearly defined. Second, the authors should consider discussing clinical and therapeutic aspect of 5HMC and other factors such as oxidative stress-induced cognitive dysfunctions into the discussion (although this suggestion by reviewer 2 is biased towards papers that he co-authored). Most importantly, this review is not a systematic (objective) review and does not follow PRISMA guidelines. It is therefore not surprising that the paper reads more like a reading note as suggested by reviewer 3. I would suggest that the authors revise their manuscript by following PRISMA methodology for paper selection and by adding a detailed description of the results of their systematic review including the metrics for including and excluding papers.

# Editor report: Decision: Exploring the epigenetic landscape: The role of 5-hydroxymethylcytosine in neurodevelopmental disorders — R0/PR3

**DOI:** 10.1017/pcm.2024.2.pr3

**Affiliations:** Institute of Cardiovascular & Medical Sciences, University of Glasgow College of Medical Veterinary and Life Sciences, United Kingdom of Great Britain and Northern Ireland

No accompanying comment.

# Editor report: Recommendation: Exploring the epigenetic landscape: The role of 5-hydroxymethylcytosine in neurodevelopmental disorders — R1/PR5

**DOI:** 10.1017/pcm.2024.2.pr5

**Affiliations:** Faculty of Medicine, University of Kiel Faculty of Medicine, Germany

No accompanying comment.

# Editor report: Decision: Exploring the epigenetic landscape: The role of 5-hydroxymethylcytosine in neurodevelopmental disorders — R1/PR6

**DOI:** 10.1017/pcm.2024.2.pr6

**Affiliations:** Institute of Cardiovascular & Medical Sciences, University of Glasgow College of Medical Veterinary and Life Sciences, United Kingdom of Great Britain and Northern Ireland

No accompanying comment.
